# Supplementary figures and images for: Broad-scale spatial distribution, microhabitat association and habitat partitioning of damselfishes (family Pomacentridae) on an Okinawan coral reef
Source: PeerJ. 2025 Feb 14;13:e18977. doi: 10.7717/peerj.18977 (PMC11831974; doi:10.7717/peerj.18977)

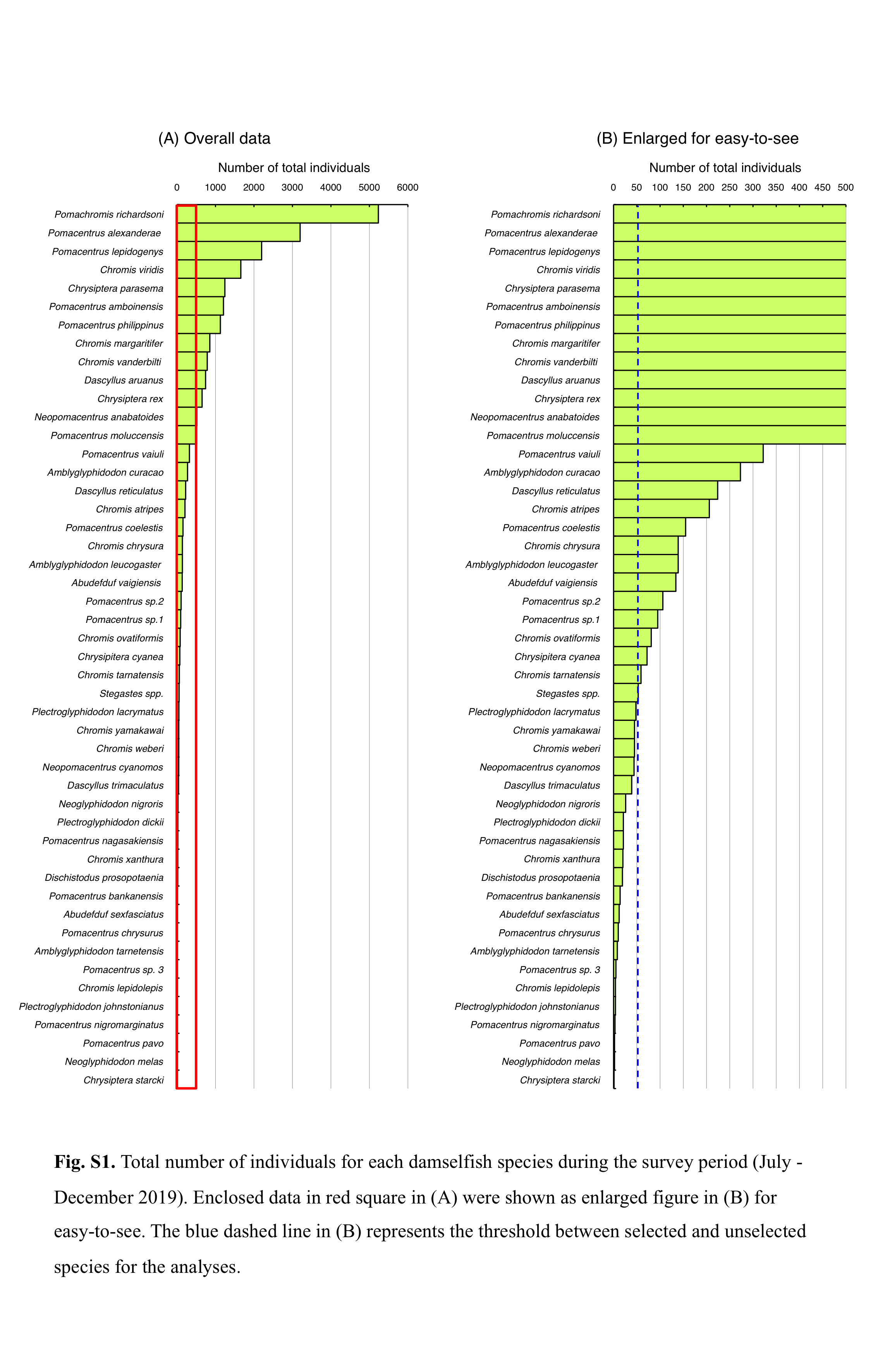

Supplement: Supplemental Information 1 — Enclosed data in red square in (A) were shown as enlarged figure in (B) for easy-to-see. The blue dashed line in (B) represents the threshold between selected and unselected species for the analyses. [file peerj-13-18977-s001.png]

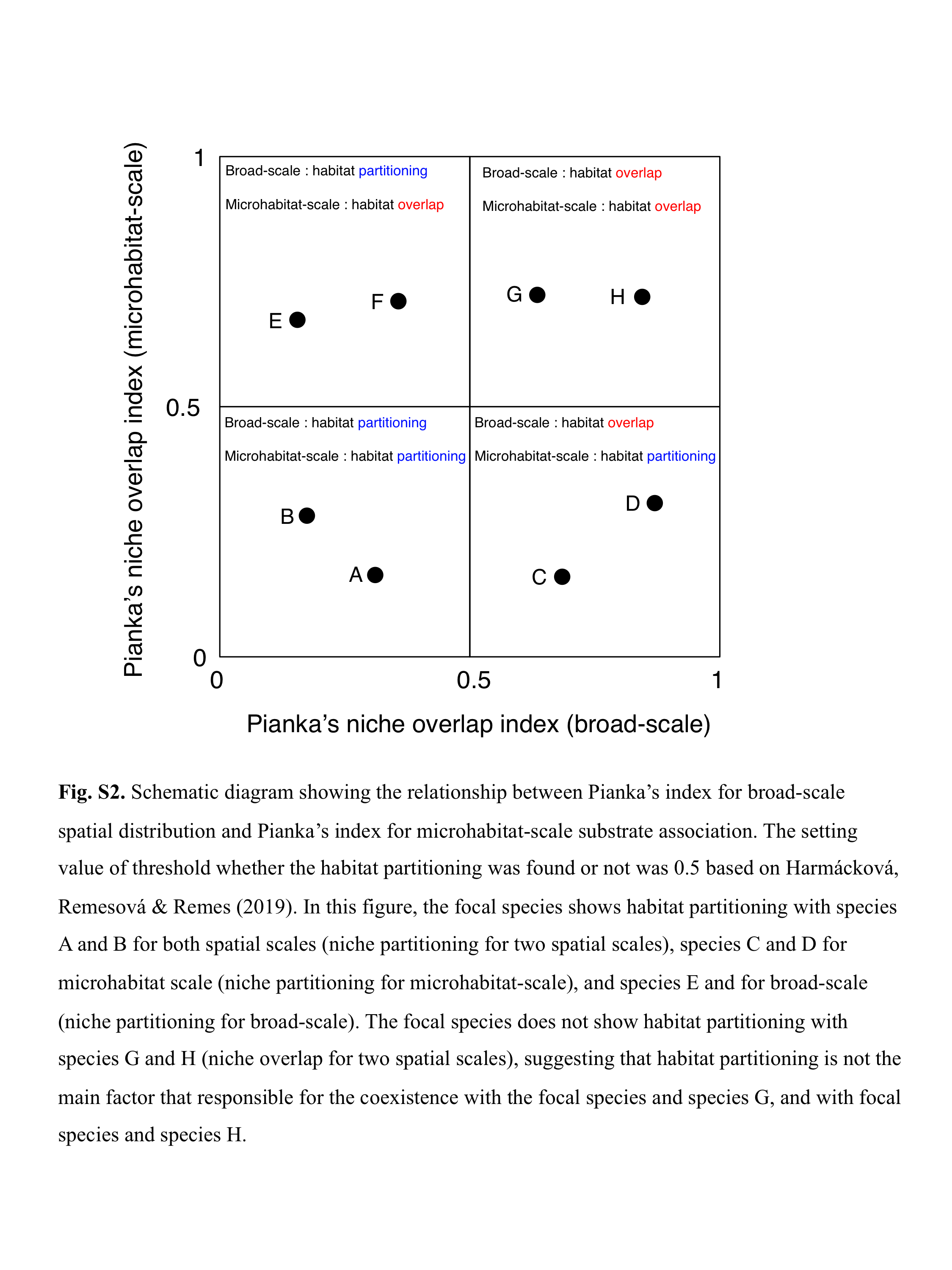

Supplement: Supplemental Information 2 — The setting value of threshold whether the habitat partitioning was found or not was 0.5 based on Harmácková, Remesová & Remes (2019). In this figure, the focal species shows habitat partitioning with species A and B for both spatial scales (niche partitioning for two spatial scales), species C and D for microhabitat scale (niche partitioning for microhabitat-scale), and species E and for broad-scale (niche partitioning for broad-scale). The focal species does not show habitat partitioning with species G and H (niche overlap for two spatial scales), suggesting that habitat partitioning is not the main factor that responsible for the coexistence with the focal species and species G, and with focal species and species H. [file peerj-13-18977-s002.png]

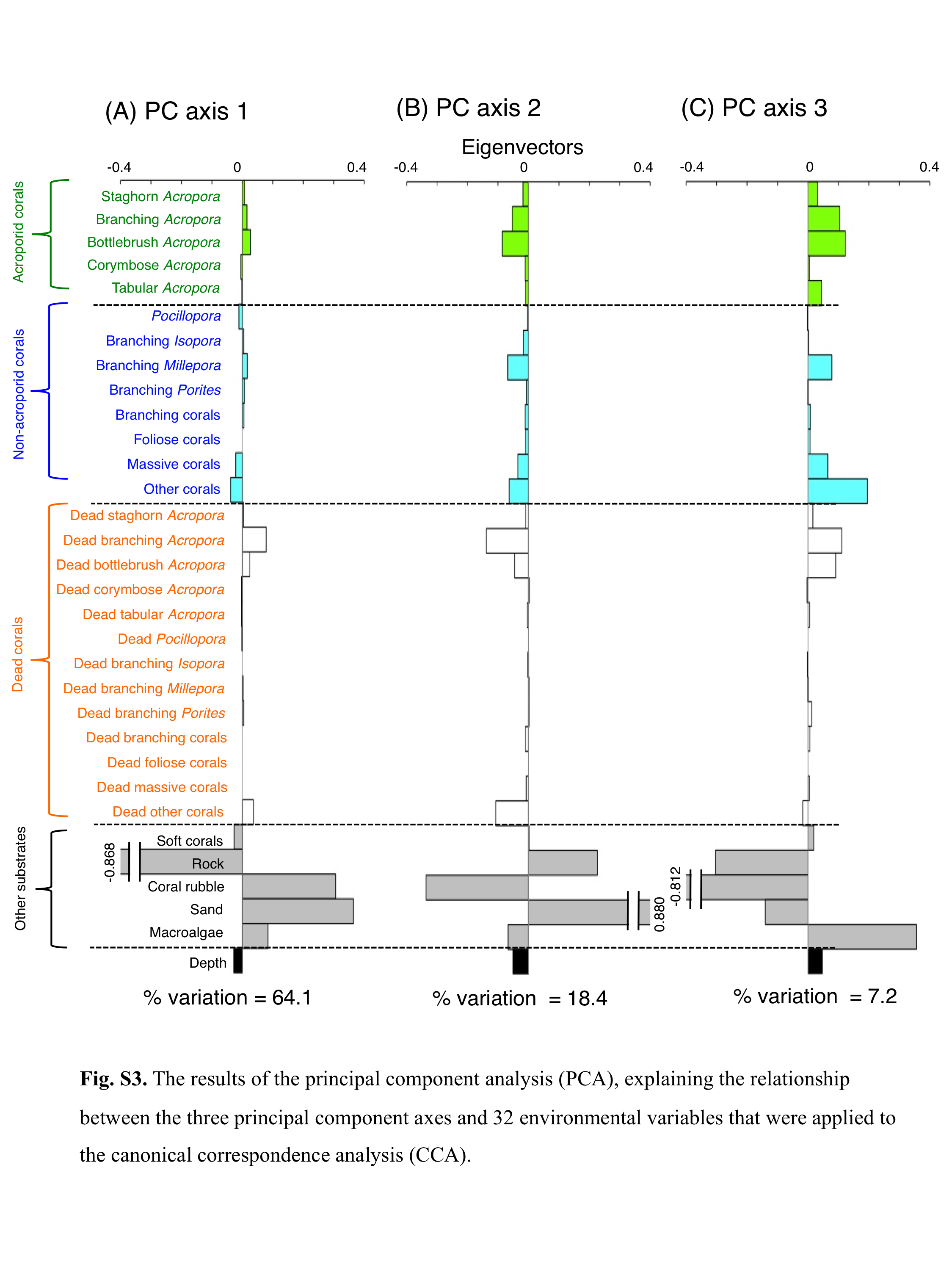

Supplement: Supplemental Information 3 [file peerj-13-18977-s003.png]

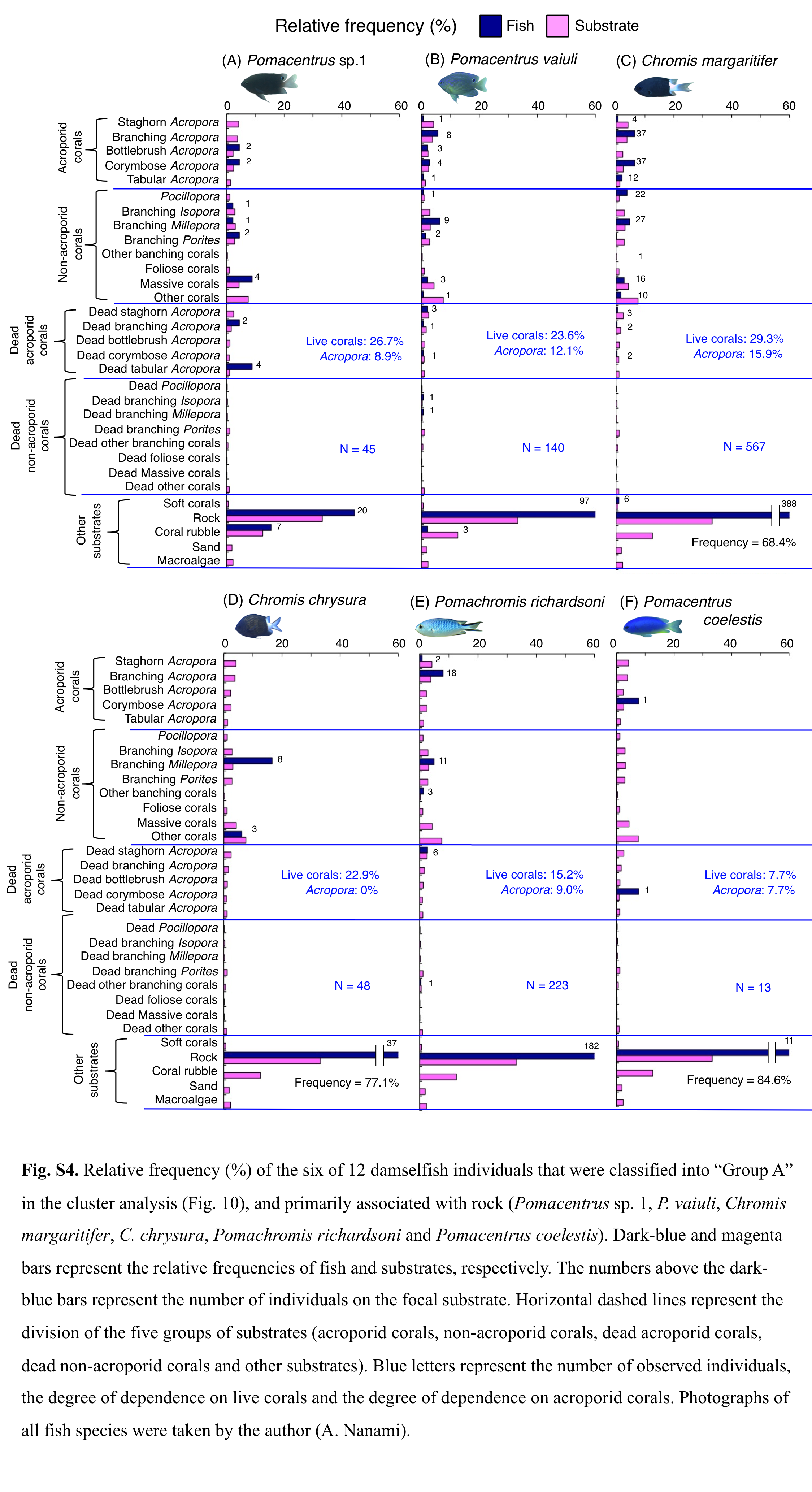

Supplement: Supplemental Information 4 — Numbers above bars represent the number of individuals on the focal substrate. Horizontal dashed lines represent the division of the five groups of substrates (acroporid corals, non-acroporid corals, dead acroporid corals, dead non-acroporid corals and other substrates). Blue letters represent the number of observed individuals, the degree of dependence on live corals and the degree of dependence on acroporid corals. Photographs of all fish species were taken by the author (A. Nanami). [file peerj-13-18977-s004.png]

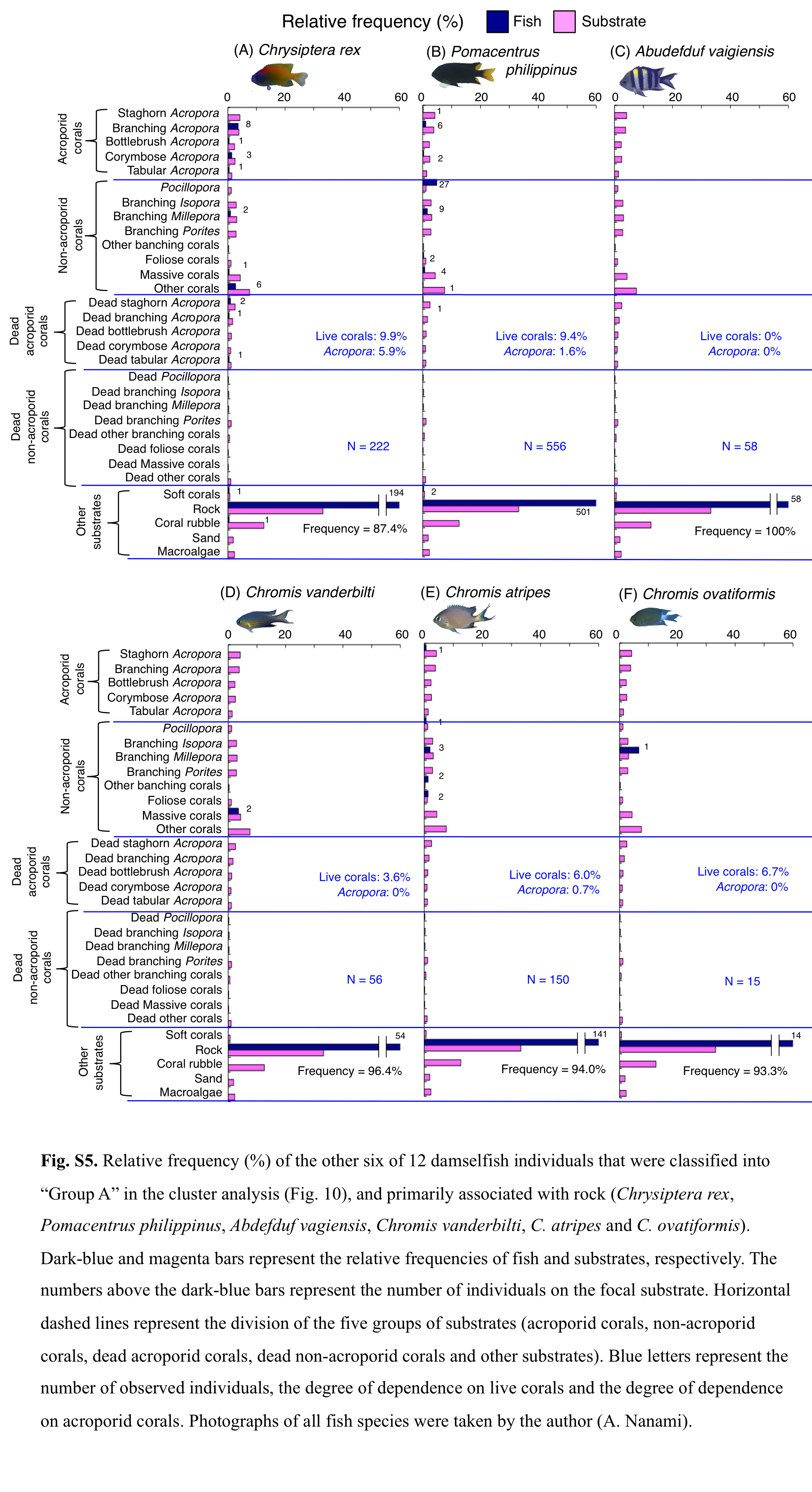

Supplement: Supplemental Information 5 — Numbers above bars represent the number of individuals on the focal substrate. Horizontal dashed lines represent the division of the five groups of substrates (acroporid corals, non-acroporid corals, dead acroporid corals, dead non-acroporid corals and other substrates). Blue letters represent the number of observed individuals, the degree of dependence on live corals and the degree of dependence on acroporid corals. Photographs of all fish species were taken by the author (A. Nanami). [file peerj-13-18977-s005.png]

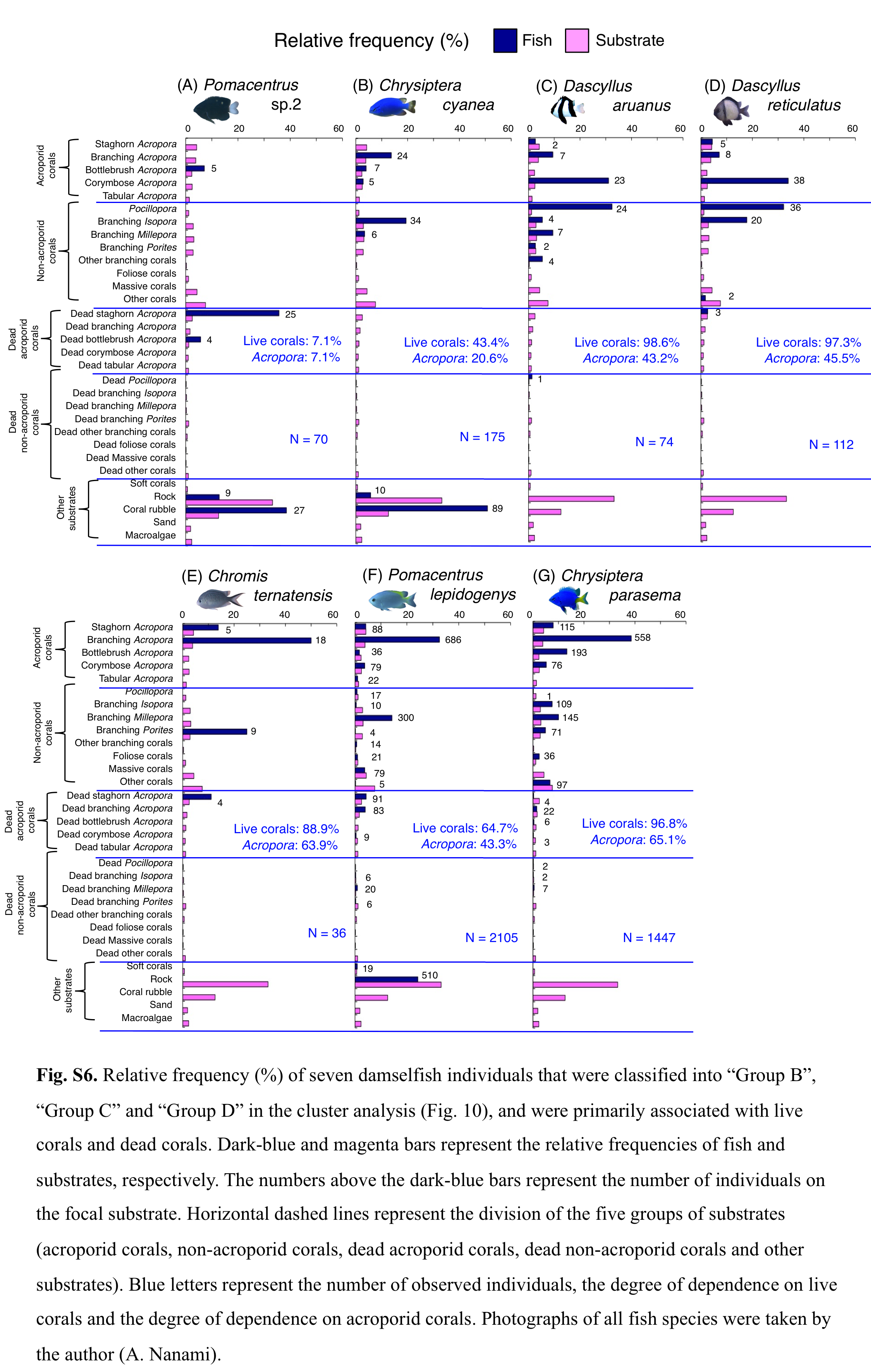

Supplement: Supplemental Information 6 — The numbers above bars represent the number of individuals on the focal substrate. Horizontal dashed lines represent the division of the five groups of substrates (acroporid corals, non-acroporid corals, dead acroporid corals, dead non-acroporid corals and other substrates). Blue letters represent the number of observed individuals, the degree of dependence on live corals and the degree of dependence on acroporid corals. Photographs of all fish species were taken by the author (A. Nanami). [file peerj-13-18977-s006.png]

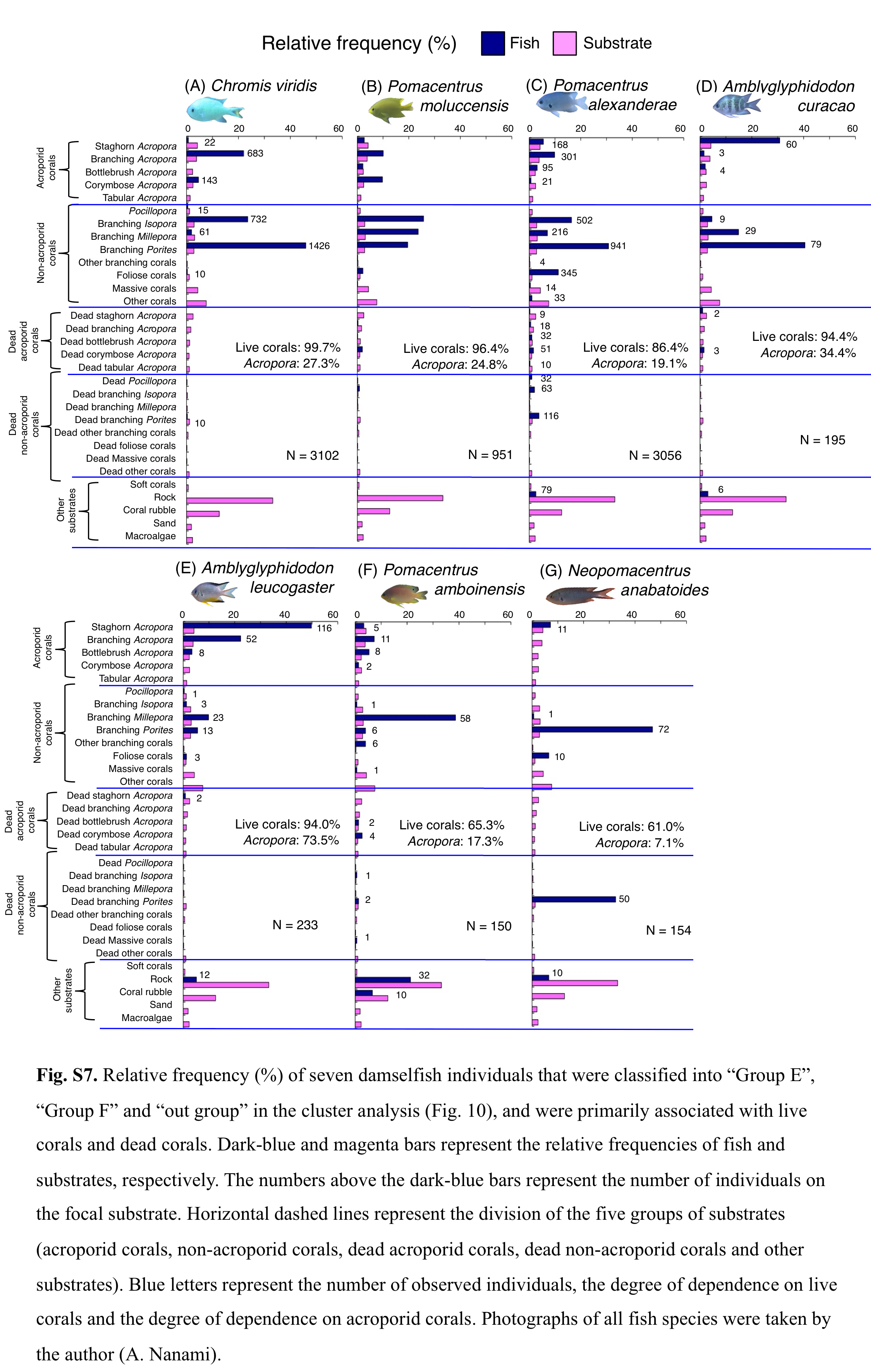

Supplement: Supplemental Information 7 — The numbers above bars represent the number of individuals on the focal substrate. Horizontal dashed lines represent the division of the five groups of substrates (acroporid corals, non-acroporid corals, dead acroporid corals, dead non-acroporid corals and other substrates). Blue letters represent the number of observed individuals, the degree of dependence on live corals and the degree of dependence on acroporid corals. Photographs of all fish species were taken by the author (A. Nanami). [file peerj-13-18977-s007.png]
